# Current Status of Western Yellow-Billed Cuckoo along the Sacramento and Feather Rivers, California

**DOI:** 10.1371/journal.pone.0125198

**Published:** 2015-04-27

**Authors:** Mark D. Dettling, Nathaniel E. Seavy, Christine A. Howell, Thomas Gardali

**Affiliations:** Point Blue Conservation Science, Petaluma, California, United States of America; Oregon State University, UNITED STATES

## Abstract

To evaluate the current status of the western population of the Yellow-billed Cuckoo (*Coccyzus americanus*) along the Sacramento and Feather rivers in California’s Sacramento Valley, we conducted extensive call playback surveys in 2012 and 2013. We also quantified the amount and distribution of potential habitat. Our survey transects were randomly located and spatially balanced to sample representative areas of the potential habitat. We estimated that the total area of potential habitat was 8,134 ha along the Sacramento River and 2,052 ha along the Feather River, for a total of 10,186 ha. Large-scale restoration efforts have created potential habitat along both of these rivers. Despite this increase in the amount of habitat, the number of cuckoos we detected was extremely low. There were 8 detection occasions in 2012 and 10 occasions in 2013 on the Sacramento River, in both restored and remnant habitat. We had no detections on the Feather River in either year. We compared our results to 10 historic studies from as far back as 1972 and found that the Yellow-billed Cuckoo had unprecedentedly low numbers in 2010, 2012, and 2013. The current limiting factor for the Yellow-billed Cuckoo in the Sacramento Valley is likely not the amount of appropriate vegetation, as restoration has created more habitat over the last 30 years. Reasons for the cuckoo decline on the Sacramento and Feather rivers are unclear.

## Introduction

The range of the western population of the Yellow-billed Cuckoo (*Coccyzus americanus*, hereafter “cuckoo”) once included riparian forests from northern Mexico to southern Canada west of the Rocky Mountains [1]. This range has contracted over the last century and currently the cuckoo maintains small breeding populations in California, Arizona, New Mexico, Texas, and northern Mexico [1, 2]. In California, breeding populations are now believed to be confined to just three areas in the state: the Sacramento River Valley, the South Fork Kern River Valley, and the Colorado River Valley [3].

Evidence suggests that the cuckoo population size along the Sacramento River has been declining over the last century [4, 5]. A decline in the cuckoo population in the Sacramento Valley was first noted by Grinnell and Miller in 1944 [6], who concluded that the loss of large areas of riparian forest was the cause of the decline. By the 1980s, 95% of riparian forest in California’s Central Valley had been lost [7]. In 1972, surveys along the Sacramento River revealed that a small population still occupied some of the remaining riparian forests [8]. Several survey efforts in the decades since have continued to find small numbers of cuckoos in the Sacramento Valley [4, 5, 9, 10].

Evidence of a population decline has motivated special status designations for the cuckoo. In 1988, California listed the cuckoo as a state endangered species [11]. In 2001, the U.S. Fish and Wildlife Service concluded that the western population was warranted but precluded from federal listing [12]. In October 2014, the U.S. Fish and Wildlife Service announced that the western distinct population segment of the Yellow-billed Cuckoo would be listed as threatened under the Endangered Species Act of 1973 (79 FR 59992).

The state listing and apparent decline in the Sacramento Valley population led government agencies and environmental organizations to include the Yellow-billed Cuckoo in their riparian forest conservation planning [13]. Since 1988, over 2,500 ha of riparian forest have been restored along the Sacramento River [14]. Most of these restorations have had many years to mature and have presumably increased the area of riparian forest for cuckoos in the breeding season.

Despite the investment in restoration in the Sacramento Valley, the current status of the cuckoo and its potential habitat has not been clearly documented. With recent vegetation mapping efforts [15] and spatial analysis tools [16], it is now possible to update the extent of potential cuckoo habitat that exists after 25 years of restoration activity.

Here, we present results from two years of surveys for Yellow-billed Cuckoos in potential habitat along the Sacramento and Feather rivers. The objectives of this study were to (1) describe the current extent and location of potential Yellow-billed Cuckoo habitat along the Sacramento and Feather rivers; (2) determine the current occupancy of habitat patches by Yellow-billed Cuckoos along the Sacramento and Feather rivers; and (3) assess these results in the context of the previous Yellow-billed Cuckoo surveys along the Sacramento and Feather rivers.

## Methods

### Ethics Statement

The Yellow-billed Cuckoo is a state protected species in California. A memorandum of understanding from the California Department of Fish and Wildlife was acquired prior to conducting our call playback surveys. Permission to conduct surveys on Sacramento River National Wildlife land was granted through a special use permit (#81627-10-0019). We did not handle any cuckoos, eggs, or nestlings and ceased call playback once a cuckoo was detected.

### Study Area

**Fig 1:**
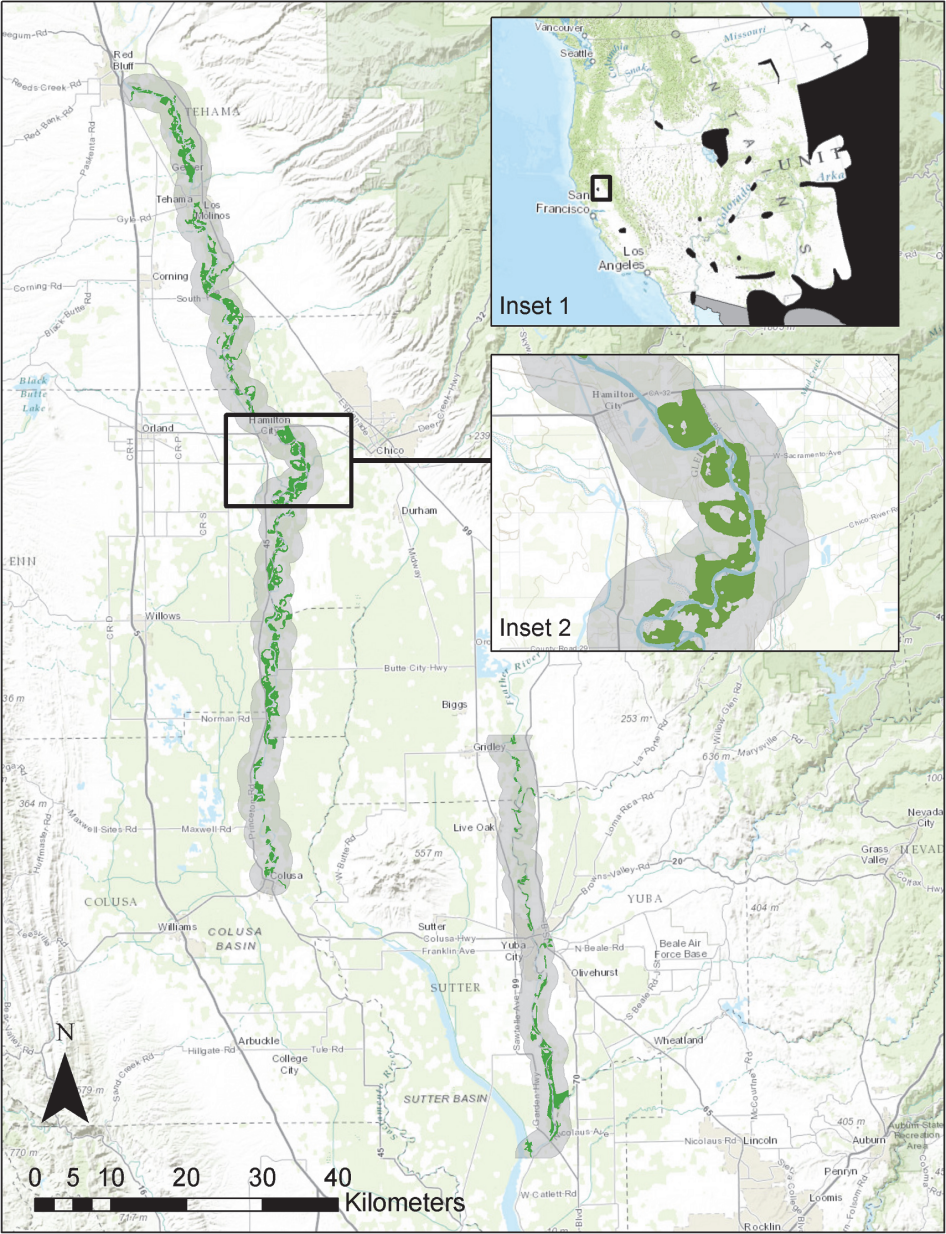
Study Area and Potential Yellow-billed Cuckoo Habitat. Study area (shaded) for Yellow-billed Cuckoo surveys along the Sacramento and Feather rivers along with potential habitat (green). Inset 1 shows study area in context of the breeding range (black) of the Yellow-billed Cuckoo in the western USA [42]. Inset 2 illustrates an example section of the study area with the potential habitat (green).

We studied Yellow-billed Cuckoos in the area along the main stems of the Sacramento and Feather rivers in California’s Sacramento Valley (Fig 1). The riparian vegetation along these rivers is surrounded by large areas of intensive agriculture (primarily fruit and nut orchards and rice fields) and smaller urban areas [17]. The area experiences hot, dry summers and mild, wet winters [18]. The hydrology of the rivers has been altered by dams upstream from the study area [19].

### Defining Potential Cuckoo Habitat

Within this study area, we limited our sampling frame to 2 km on either side of the Sacramento (Red Bluff to Colusa; ca. 100 river miles) and Feather (Gridley to Nicolaus; ca. 32 river miles) rivers (Fig 1). Although riparian forest does exist outside the study area, the majority of cuckoo sightings during the breeding season have been <2 km from the Sacramento or Feather rivers [5]. Within this area, we needed to define and quantify potential cuckoo habitat to (1) refine the sampling frame for our survey effort and (2) describe the extent and distribution of potential habitat.

Not all land cover within our sampling frame is potential cuckoo habitat. We refined this area using information about the patch configuration of riparian vegetation. In California, cuckoos are riparian forest obligates that will use relatively young forests (e.g., 4 years old; [10]) as well as mature stands [8], and may prefer areas with both [20]. To define potential habitat, we used the Central Valley Riparian Mapping Project vegetation layer (medium-scale layer, 2009 imagery; [15]) to identify and map all riparian vegetation (including restoration sites) within 2 km of the river. The vegetation classifications used to define potential habitat were “RWF—Riparian Evergreen and Deciduous Woodland” and “RWS—Southwestern North America riparian wash/scrub”, which captured a wide array of riparian vegetation alliances (Table 1).

**Table 1:** Vegetation types [43] that characterize the riparian classifications chosen to define potential Yellow-billed Cuckoo habitat.

Classification	RWF	RWS
**Vegetation types**	*Acer negundo* alliance	*Baccharis salicifolia* alliance
	*Juglans hindsii* stands	*Cephalanthus occidentalis* alliance
	*Platanus racemosa* alliance	*Rosa californica* alliance
	*Populus fremontii* alliance	*Salix exigua* alliance
	*Quercus lobata* alliance	*Salix lasiolepis* alliance
	*Salix gooddingii* alliance	*Sambucus nigra* alliance
	*Salix laevigata* alliance	

RWF = Riparian Evergreen and Deciduous Woodland, RWS = Southwestern North American riparian wash/scrub.

Potential Yellow-billed Cuckoo habitat is best defined as patches of riparian vegetation with sufficient area (>15 ha) to support cuckoos [2]. Many patches of riparian vegetation are likely too small to provide habitat for cuckoos [8, 9, 21]. Some areas with other land covers (e.g., grasslands) that are surrounded by or between patches of riparian forest and scrub can be incorporated into cuckoo territories and are not necessarily barriers [5].

To quantitatively define potential cuckoo habitat, we used the program PatchMorph [16] in ArcGIS (ESRI, Redlands, California). This program delineates patches using an input GIS layer of riparian vegetation (described above) and user defined organism-specific parameters of minimum patch size, minimum patch width, and maximum width of gaps in suitable habitat (see S1 Appendix for the PatchMorph parameters used in this study; note that the minimum patch size was applied in ArcGIS after PatchMorph was run with no minimum). Previous efforts to define cuckoo habitat on the Sacramento River defined the minimum patch size as 5 ha, the minimum patch width as 100 m, and the maximum gap width as 100 m [21]. In this study, we used a minimum patch size of 15 ha, with the same values for minimum patch width and maximum gap width. We chose to increase the minimum patch size to better reflect the most recent estimate of minimum cuckoo home-range size derived from radio telemetry studies in Arizona (15 ha; [22]).

Our final sampling frame represented potential habitat patches of suitable riparian vegetation within 2 km of the Sacramento or Feather rivers (Fig 1).

### Field Survey

#### Sampling design

Due to logistical and funding constraints, we were unable to survey all the potential cuckoo habitat. Instead, we generated a sampling design to survey a subsample of the area which was used to make inferences over the entire area. Our sampling design was based on a 300 m grid (developed from the 100 m Military Grid Reference System) overlaid on the habitat patches. The size of the grid cells (9 ha) was smaller than the smallest patch, so each patch was covered by multiple cells, but large enough that cells near each other could be chosen and non-overlapping survey transects established.

We randomly selected grid cells from which to start survey transects (see below) using a generalized random-tessellation stratified (GRTS) sampling design [23, 24]. The GRTS sampling method is increasingly being adopted for large-scale environmental monitoring programs, in part because it creates a spatially balanced random sample, while being flexible enough to allow additional samples to be added or removed without compromising the spatial balance of the overall sample [23]. We used the GRTS algorithm to select starting grid cells and “oversample” locations for both rivers. These oversamples were used to choose extra starting grid cells if time and staffing allowed and to replace random sites that needed to be dropped due to access restrictions or other logistical constraints. A different set of starting grid cells was chosen each year. In 2012, 54 starting grid cells were chosen (44 on the Sacramento River and 10 on the Feather River) and in 2013, 61 starting grid cells (51 on the Sacramento River and 10 on the Feather River).

From each starting grid cell, we established a transect of points at which surveys were conducted. Transects started with a survey point in the GRTS chosen grid cell and continued with survey points spaced at regular intervals through the potential habitat patch until the entire patch had been thoroughly surveyed or the surveyor ran out of time. Transect points were established based on logistical feasibility (ability to move through the site in a timely manner; able to complete in one morning before noon) and in such a way that as much of the patch as possible was covered. Transects ranged from 8 to 34 points. If the patch was entirely covered with time to spare, and another patch was within 500 m and on the same side of the river, the surveyor moved to that patch and surveyed until they ran out of time.

#### Survey protocol

Surveys were conducted using a call playback protocol developed by Halterman et al. [25] and adopted by the Western Yellow-billed Cuckoo Working Group. Within a transect, survey points were spaced approximately every 100 m, and five sets of calls (with one minute of silence in between) were played at each point. A recording of a Yellow-billed Cuckoo (provided by M. Halterman) consisting of a contact call (series of “kuks” and “kowlps”) was broadcast using an iPod Nano digital music player (Apple, Cupertino, California) and a Big Horn Remote speaker (Cass Creek, Grawn, Michigan). The volume was set to produce ~70 decibels at 1 m, allowing the call to be heard at least 100 m away through vegetation. When a cuckoo was detected, we stopped the call playback and recorded detection information. Points with a detection in one round were surveyed again in subsequent rounds. After a cuckoo detection, the surveyor moved 300 m (skipping points) and resumed the survey to avoid further disturbing or attracting the previously detected individual. If that individual responded again, the surveyor moved further (300 m or more depending on cuckoo behavior) before resuming the survey.

It is possible that after a cuckoo was initially detected, it could be detected again that same day even after the surveyor moved 300 m as specified by the protocol. To address this issue, surveyors determined if subsequent detections were of the same bird or another individual. To do this, surveyors estimated the location of the cuckoo, determined if and where it moved during the observation, and observed habitat characteristics in an effort to keep track of the detected cuckoo. All surveyors were trained in the protocol prior to conducting surveys. During the study there were 8 occasions (5 in 2012, 3 in 2013) where the surveyors determined that one or more cuckoo detections on a transect were redetections of an individual that had been detected earlier that morning. These same day redetections were not included in any analyses.

All of the transects were surveyed four times, with each visit separated by at least 12 days but no more than 20 days. The survey period was from 15 June to 16 August, corresponding with the height of breeding activity [1]. Our surveys were not designed specifically to find nesting activity, but we did attempt to follow individuals for ~30 minutes after their initial detection to record any evidence of breeding.

We added two specifications to the Halterman et al. [25] protocol to aid in our planned statistical analysis. First, we surveyed the same points during each visit to a transect, whereas the protocol only requires the transects to be surveyed, not individual points. Second, initial starting points for transects were randomly chosen (see above).

### Statistical Analysis

We used 500 m grid cells as the analysis unit to summarize our results. This size analysis unit (25 ha) is a reasonable approximation of the average size of a cuckoo territory [22]. The grid cells defining the analysis units were derived from the 100 m Military Grid Reference System and those units that contained any amount of potential habitat were included. We chose to analyze the Sacramento and Feather rivers separately because they have been treated separately during previous survey efforts. We analyzed survey data from 2012 and 2013 separately.

#### Naïve occupancy

We calculated the naïve occupancy, which was the percent of surveyed analysis units with at least one detection during the four survey rounds. This method does not account for probability of detection, and hence underestimates true occupancy if detectability is less than perfect.

#### Estimating occupancy for the entire survey area

The naïve occupancy was then applied to the analysis units that were not surveyed to estimate occupancy for the entire survey area. This method relies on our random sample to capture the variability of potential habitat within each analysis unit. More sophisticated occupancy analyses could not be performed due to the paucity of second detections within an analysis unit in a single year.

### Comparison to Previous Surveys

Since the early 1970s several comprehensive surveys of the Sacramento and Feather rivers have been conducted. We compiled the reported numbers of Yellow-billed Cuckoos found along the Sacramento and Feather rivers by these previous researchers (S2 Appendix).

We gathered information on survey effort and results published in journal articles, graduate theses, and reports to government agencies (S2 Appendix). In an effort to standardize the results of these studies which differ in survey protocol and effort, we calculated the number of cuckoos detected per surveyor day (one person surveying one day). Although the number of hours that were surveyed would be a more accurate account of effort, the majority of studies did not report total survey hours. In addition to differences in reporting effort, there were also differences in how the number of cuckoos detected was reported. In some cases, the total raw number of cuckoo detections was included while in others the total number reported was modified after some interpretation by the researchers. For example, in some studies certain types of call responses were interpreted as representing a paired cuckoo or an unmated individual (and ultimately reported as two or one respectively), an interpretation which was recently found to be unreliable [22]. Hence, to standardize detections for use in comparisons across surveys, when the raw number of detections were not reported we used the most conservative estimate of detections by counting reported pairs as one detection and unmated birds as one detection. When a range of pairs and/or unmated birds was given we chose the low end of each.

## Results

### Potential Habitat

**Fig 2:**
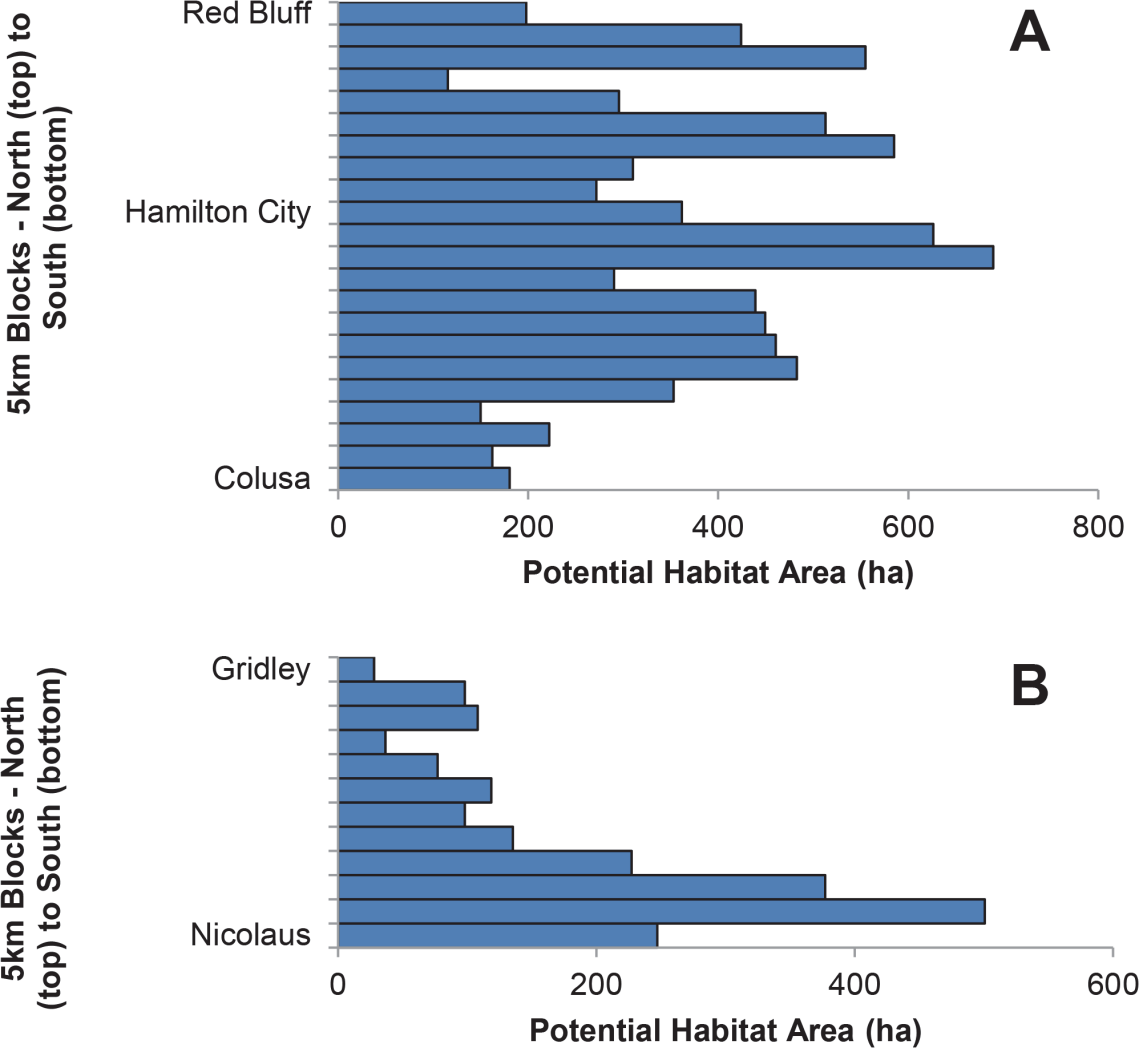
Distribution of Potential Yellow-billed Cuckoo Habitat. Hectares of potential Yellow-billed Cuckoo habitat from north to south in 5 km increments along the (A) Sacramento and (B) Feather rivers, CA with city names as reference points.

We identified a total of 84 potential habitat patches along the Sacramento River and 31 along the Feather River (Fig 1, S1 GIS Files). The potential habitat patches averaged 97 ha (range 15–555 ha) along the Sacramento River and 66 ha (range 17–476 ha) along the Feather River. The total area of potential habitat was 8,134 ha along the Sacramento River and 2,052 ha along the Feather River, for a total of 10,186 ha. Potential habitat was distributed relatively evenly across the entire Sacramento River study area, though the northern half seems to have sections with the largest areas (Fig 2A). Along the Feather River, most of the potential habitat was in the southern portion of our study area (Fig 2B).

### Survey Effort

Survey effort was similar between 2012 and 2013 (Table 2). There was a slight increase in the area covered in 2013 due to a larger field crew. Transects were surveyed four times during the survey period. A small number of individual points were surveyed fewer than four times because they were skipped due to a nearby cuckoo detection, predator observation, or excessive noise (e.g., farm equipment).

**Table 2:** Yellow-billed Cuckoo survey effort along the Sacramento and Feather rivers, 2012–13.

	Sacramento River		Feather River	
Survey Effort	2012	2013	2012	2013
**Transects**	44	51	10	10
**Points**	1100	1283	288	270
**Person hours**	875	845	207	179
**Surveyor days**	182	204	40	40
**Surveyed potential habitat (ha)**	2571	2902	643	573

Area of potential habitat surveyed assuming the call playback elicits a response within an average of 150 m radius of the survey point.

The area of potential habitat we surveyed depends on the effective range of our broadcast call. The volume of the broadcast call was set high enough to be heard at least 100 m through thick vegetation, therefore it could be heard further in thinner vegetation. We estimated that on average our broadcast call covered a circle with a 150 m radius. Based on this radius, over the two years we surveyed 3,958 ha (48.7% of the total) of potential habitat on the Sacramento River and 862 ha (42.0% of the total) of potential habitat on the Feather River.

### Number of Cuckoos

**Fig 3:**
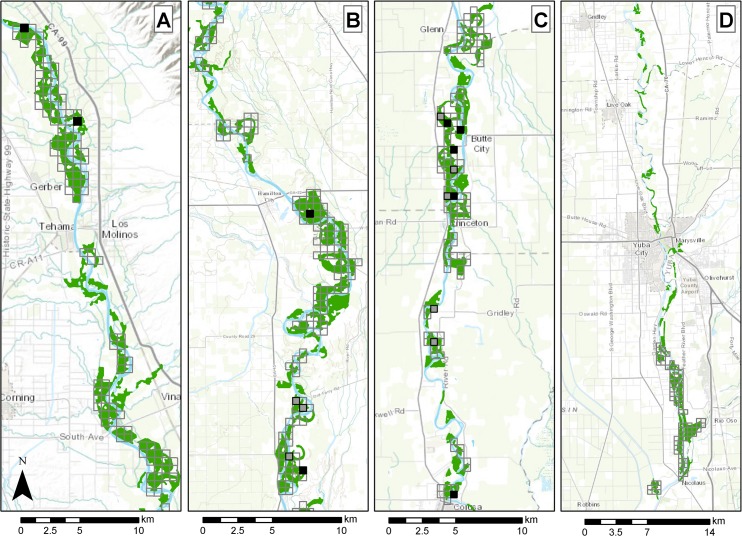
Yellow-billed Cuckoo Survey and Detection Locations. Locations of Yellow-billed Cuckoo surveys during our study (gray outlined boxes) and detections during surveys in 2012 (filled gray boxes) and 2013 (black boxes), along the Sacramento River (panels A, B, and C from north to south) and Feather River (panel D), CA. Green areas represent potential Yellow-billed Cuckoo habitat. No cuckoos were detected along the Feather River during either year.

Along the Sacramento River, we detected Yellow-billed Cuckoos on 8 occasions in 2012 and 10 occasions in 2013. Each year there was one detection in restored riparian forest as well as one detection in narrow remnant riparian forest with adjacent restored forest. Detections spanned the length of the study area, though most of them were in the southern half (Fig 3). Only two of the detections were along a transect with a previous detection during the same year.

Naïve occupancy rates of approximately 3% along the Sacramento River were similar between years (Table 3). Applying the naïve occupancy rate to all potential habitat in our sampling frame, we estimate that 27–28 analysis units were occupied. If we assume that each occupied analysis unit represents a mated pair of birds, this would suggest a population of under 30 pairs.

**Table 3:** Naïve occupancy estimates for Yellow-billed Cuckoos in surveyed analysis units for 2012 and 2013.

	Sacramento River		Feather River	
	2012	2013	2012	2013
**Naïve occupancy rate**	0.032	0.033	0	0
**Estimated # of occupied analysis units**	26.9	27.5	0	0

Estimate of occupied analysis units based on all 500 m grid cells that contain at least some potential habitat.

Because there were no detections along the Feather River, we cannot estimate occupancy. We have no evidence to indicate that cuckoos continue to occupy potential habitat along the Feather River.

### Comparison to Previous Surveys

**Fig 4:**
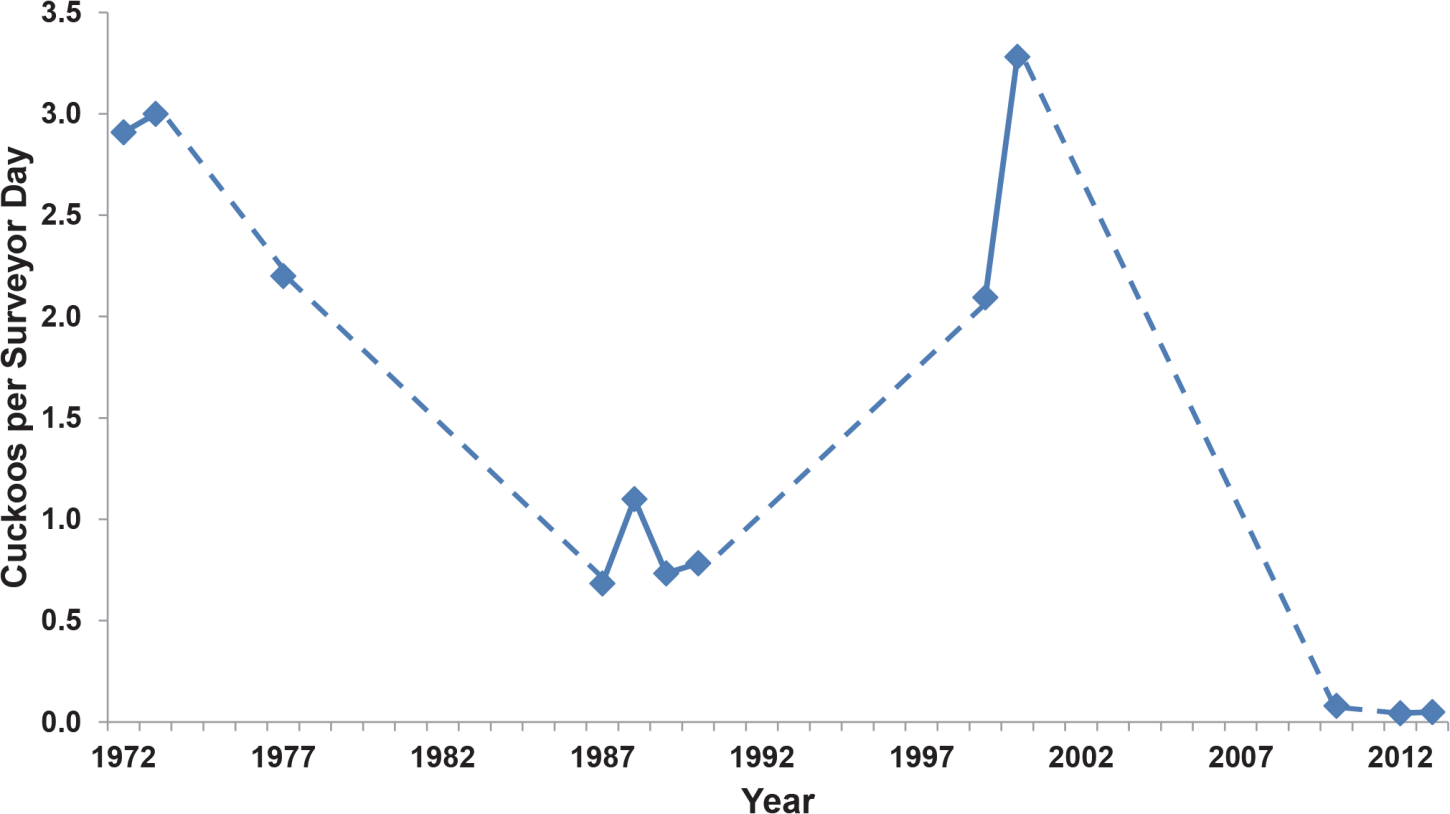
Yellow-billed Cuckoo Detection Rate, 1972–2013. Number of Yellow-billed Cuckoo detections per surveyor day for surveys along the Sacramento River, 1972–2013. Dashed lines connect non-consecutive survey years.

Our surveys showed substantially lower detections per surveyor day than any other previous study along the Sacramento River (Fig 4, S2 Appendix). Our rate of detection was 15–75 times lower than previous surveys. Two of the previous five surveys along the Feather River detected small numbers of cuckoos, with the other three surveys having zero detections (S2 Appendix).

## Discussion

Overall, our results suggest that despite significant efforts to increase the amount of available habitat for Yellow-billed Cuckoos along the Sacramento and Feather rivers, this increase has not resulted in an increase in the population. Given that habitat restoration has resulted in positive responses for riparian birds and other taxa in the Central Valley including the Sacramento River [14, 17, 26], the lack of response by Yellow-billed Cuckoos suggests that something other than amount of breeding habitat is responsible.

### Potential Habitat

Over the last three decades, there have been more than 2,500 ha of riparian forest restored along the Sacramento River [14] and more than 400 ha along the Feather River (H. Swagerty personal communication). One of the objectives of these restoration efforts was to create more Yellow-billed Cuckoo habitat [13]. These efforts have increased the amount of potential cuckoo habitat, and today there are 8,134 ha of potential habitat along the Sacramento River and 2,052 ha along the Feather River.

Understanding how much available habitat has changed over the last 50 years is complicated because previous efforts have used different definitions of potential habitat. There have been two previous efforts to estimate the amount of potential cuckoo habitat in the Sacramento Valley. In the early 1970s, Gaines [27] used topographical maps and aerial photographs to estimate there was 1,073 ha of potential cuckoo habitat along the Sacramento River and 121 ha along the Feather River. This analysis was based on the amount of “uncultivated woody vegetation” that was greater than 100 m wide and 10 ha in area. More recently, Girvetz and Greco [21] used vegetation data from 1997 and 1999 with PatchMorph to estimate there was 6,018 ha of potential cuckoo habitat along the Sacramento River.

While our estimate is larger than both of these estimates, a direct comparison is difficult because ours included patches further from the river reflecting a more recent recognition that cuckoos will use areas > 100 m from the river [5], and because we used a larger minimum patch size (15 ha instead of 5 or 10 ha) than either of these analyses, which reflects more recent information on the home range size of cuckoos [22].

In the Riparian Habitat Joint Venture Riparian Bird Conservation Plan [13], ecologists recommended that 6,070 ha of Yellow-billed Cuckoo habitat on the Sacramento River and 1,012 ha of habitat on the Feather River was necessary to support a self-sustaining population, assuming ~40 ha per pair [28]. Their definition of suitable habitat was willow-cottonwood forest of any age that was greater than 100 m in width and 20 ha in area. It should be noted that our estimate included forest patches as small as 15 ha and forest types other than willow-cottonwood, and therefore comparisons should be made with care. In the future, we suggest that revisions to the amount of potential cuckoo habitat should use PatchMorph [21] with the parameters we used in this analysis (S1 Appendix) and updated vegetation layers. Nonetheless, the amount of currently available potential habitat suggests that restoration efforts have been effective at creating the vegetation community considered important for increasing the number of Yellow-billed Cuckoos.

The successful restoration of appropriate cuckoo habitat is underscored by recent findings that cuckoos are using restored riparian forest. In this study, 2 of our 18 cuckoo detections were in areas that had been restored (10–11 years old). In the 2010 surveys, 8 of the 23 detections were in areas restored 6–15 years prior [29]. Surveys of restored areas 4–18 years old along the Sacramento River in 2007–2008 had 10–15 detections [10]. Riparian restoration of several age classes have been effective at creating areas where Yellow-billed Cuckoos are now detected and hence suggests that the amount of potential habitat has greatly increased.

### Occupancy of Potential Habitat

In both years of the study, we detected Yellow-billed Cuckoos at ~3% of the analysis units we surveyed. Applying this estimate to the entire sampling frame (833 analysis units), we would expect a total of 27 analysis units with detections. If we assume that a detection within an analysis unit represents a pair, this would mean that the population is no more than 27 pairs, which is below the Riparian Habitat Join Venture management goal of 150 pairs [13, 28].

This estimate should be interpreted in light of two critical uncertainties. First, by using naïve occupancy, we assume perfect detection. If detection is less than perfect, which it very likely is, then our approach would be an underestimate of the true number of pairs. In Arizona, cuckoos had a detection probability of 32% using the same survey protocol [22]. One approach to accounting for imperfect detection is occupancy modeling [30]. Our survey approach, with four visits to each site, was designed to allow us to perform an occupancy analysis following MacKenzie et al. [30]. However, this analysis method relies on repeated detections over subsequent visits to develop a probability of detection and the very low number of detections on subsequent surveys prevented us from using this approach.

The second uncertainty is the assumption that all detections represent breeding pairs. We believe this assumption is highly unlikely along the Sacramento River given the very low number of analysis units where birds were detected more than once. Both mated and unmated cuckoos have been shown to respond to call-playback surveys with mated cuckoos detected at a higher rate [22]. Furthermore, we could not confirm the pair status of any of the cuckoos we detected since we did not observe nesting activity (e.g., cuckoos carrying nest material or food) or more than one cuckoo at any single location.

### Comparison to Previous Surveys

Estimating trends in Yellow-billed Cuckoo populations in the Sacramento Valley from historical survey efforts is complicated because these efforts have varied in protocol, effort, habitat sampled, and interpretation of responses. Since 1972 there have been 12 surveys of the Sacramento River and 5 surveys of the Feather River (S2 Appendix). Some of the studies reported a population estimate based on available habitat and estimated territory size, while others assumed their surveys detected all of the cuckoos. An estimate of 120 pairs along the Sacramento River in 1972 was calculated by assuming all of the estimated 1,200 ha of potential cuckoo habitat was occupied at a density of one pair per 10 ha [8]. That 1972 population estimate was revised to 60–96 pairs using a naïve occupancy of 60–80% for the 1,200 ha of habitat [9]. Surveys of the Sacramento River in 1977 found 29 pairs and estimated up to 60 pairs using a naïve occupancy of 50% [9]. Halterman [4] did not estimate a Sacramento River population size but instead reported that in the habitat surveyed (73 total sites) there was a population of between 18 pairs (along with 23 unmated) and 35 pairs (31 unmated) from 1987–1990. Similarly from 1999–2000 between 28 and 40 cuckoo pairs were found along the Sacramento River [5]. Although the 1987–1990 and 1999–2000 surveys were similar in extent, the earlier surveys were conducted by playing calls every 200 m while the later surveys used a distance of 100 m and had surveyors move 300 m from a cuckoo detection before resuming surveys. These differences may have the effect of increasing the number of detections on the later surveys simply due to the protocol. Our naïve occupancy estimate of 28 pairs is lower than most previous estimates and suggests a decline since the earliest surveys, even under a generous assumption that all detections represented pairs of cuckoos.

However, because estimating the number of breeding pairs of cuckoos relies on a number of assumptions, an alternative approach is simply to evaluate the number of detections standardized by the amount of effort. In our analysis of historical surveys, detections per surveyor day decreased from ~3 in early surveys to ~0.05 in our current surveys (Fig 4). Although this method does not account for differences in protocol such as length of playback, the severe decline in detections, despite a substantial increase in effort and potential habitat surveyed, strongly suggests that the Yellow-billed Cuckoo is at-risk of extirpation in the Sacramento Valley. Further, by the time surveys began in the 1970s the cuckoo population was apparently already greatly reduced [8] making a historic decline of even greater magnitude.

Another important difference between our surveys and historical ones is the method for choosing where to survey. Our use of potential habitat defined by remotely-sensed vegetation data and the PatchMorph program in combination with the GRTS sampling method explicitly defines how survey sites were chosen. In 1972, survey sites were chosen “to sample the range of available habitat” [8], and in 1977 they were chosen based on “where the cuckoo has been reported in the past or where habitat appeared to meet the requirements of the species” [9]. The more recent surveys in 1999 and 2000 focused on “public access riparian areas in California known or suspected to support breeding populations of Yellow-billed Cuckoos in the last 25 years” [5]. Future surveys should clearly define how survey sites were chosen and avoid only surveying where cuckoos have been found or where habitat appears suitable.

The decline of cuckoo numbers along the Sacramento River is unfortunate in light of the decades of investment to create additional habitat. Indeed our naïve occupancy analysis suggests that up to 97% of the potential habitat is unoccupied. In contrast, the Southern California population along the Lower Colorado River has remained stable despite substantial increases in the amount of restored riparian forest [31]. For this area, the pattern has been for an increase in use of restored sites and a reduction in use of remnant sites [31]. The use of restored forests by cuckoos shown by our work and that of others [10, 31] is encouraging despite stable or decreasing populations.

### Possible Reasons for Decline

The current limiting factor for the Yellow-billed Cuckoo in the Sacramento Valley is likely not the amount of appropriate vegetation, as there has been a net gain over the last 30 years. Thus, the cause of the continued decline of the population in the Sacramento Valley remains unknown. The decline may be an artifact of the habitat conversion that occurred over the past 150 years which left less than 5% of the historic riparian forest [7]. In other words, with such a dramatic loss of habitat, the amount of forest restored may not be enough to slow the decline already in motion. For example, habitat loss may have reduced the cuckoo population to a level at which Allee effects impacted the ability of the population to recover [32, 33].

Another possible contributor to the decline is the condition of food resources. Cuckoos are often observed feeding at outbreaks of caterpillars and large insects [1]. If these insects are less abundant now and/or affected by agricultural pesticides then the reduction in food resources could be a driving factor, especially for juvenile survival, despite an increase in riparian habitat. The impact that pesticides have on prey availability for other insectivorous birds that use habitat in a matrix of agriculture is of increasing concern [34–36]. However, given that many other species of insectivorous birds are known to be significantly increasing in restored and remnant forests in the Sacramento Valley [17], this explanation would require that the impact of pesticides on food resources for Yellow-billed Cuckoos is substantially different then the effect on prey items of other insectivorous birds.

It is also possible that changes on the wintering grounds and/or during migratory stopovers are impacting the population of cuckoos breeding in the Sacramento Valley either directly by increased mortality in these life stages or indirectly by carry-over effects as has been noted for other migratory species [37, 38]. The Yellow-billed Cuckoo spends only 3–4 months in the Sacramento Valley with the rest of the year spent in transit to or on its wintering grounds in South America. Recent studies have begun to shed light onto this part of the cuckoo’s life cycle, with some surprising results like late summer movements into Mexico [39, 40] and different spring and fall migration pathways [40].

Because none of these reasons for decline are mutually exclusive, we believe the most fruitful area of conservation research lies in developing a greater understanding of the full-life cycle of Yellow-billed Cuckoos. Currently, the most important step in that process would be to develop a better understanding of their migratory connectivity, including identifying migration stopover and wintering sites, and studying the cuckoo’s ecology in those locations.

### Management Implications

Our Yellow-billed Cuckoo population estimates for the Sacramento and Feather rivers are well below the targets set by the Riparian Habitat Joint Venture, though our estimates of potential habitat are higher than the targets. Despite the apparent lack of response to restoration for this cuckoo, we still suggest additional restoration is warranted. If limiting factors are eased, and the population begins to recover, it will be essential to have adequate breeding habitat. Additionally, restoration has been demonstrated to increase populations of several species of resident and migratory birds in the Sacramento Valley, so benefits extend beyond the cuckoo [17] especially in areas with extensive riparian forest within the greater landscape [41]. Hence, with cuckoos shown to use restored forests (as soon as four years after planting), we recommend continued restoration of riparian forests throughout the Sacramento Valley focusing on areas adjacent to existing forest.

## Supporting Information

S1 Appendix Inputs into program PatchMorph for Yellow-billed Cuckoo potential habitat delineation. (DOCX)

S2 Appendix Historic Yellow-billed Cuckoo Survey Results and Effort for the Sacramento and Feather Rivers. Historic to current Yellow-billed Cuckoo survey studies along Sacramento and Feather rivers, 1972 to 2013. Information included is the number of cuckoos reported, number of rounds of surveys per breeding season, citation, number of surveyor days (the total number of days a survey was conducted by each person conducting surveys), range of survey dates, our conservative estimate of the number of cuckoo detections (derived by assuming any reported pair was only single a detection of one individual and each unmated detection was one individual), and the number of cuckoos per surveyor day. The 2007 and 2008 study on the Sacramento River only surveyed restored habitat within the Sacramento River National Wildlife Refuge and hence was not used in the analysis of change.
(DOCX)

S1 GIS Files Potential Habitat PatchMorph Output Shapefiles for the Sacramento and Feather Rivers. (ZIP)
