# Supplementary material for: Current Status of Western Yellow-Billed Cuckoo along the Sacramento and Feather Rivers, California
Source: PLoS One. 2015 Apr 27;10(4):e0125198. doi: 10.1371/journal.pone.0125198 (PMC4411113; doi:10.1371/journal.pone.0125198)
Supplement: S1 Appendix — (DOCX) [file pone.0125198.s001.docx]

**S1 APPENDIX. Inputs into program PatchMorph for Yellow-billed Cucko potential habitat delineation.**

PatchMorph inputs for the Sacramento River.

Run name = Sac2

Input landcover map = Sac_RWFRWS

Landcover map field = YBCU_HAB

Cell size = 10

Density Filter

Density filter = 0

Density neighborhood = 5

Density threshold = 0.25

Hard Barrier

Hard barrier = 1

Hard barrier map = Sac_RWFRWS

Hard barrier map field = WATER

Habitat Suitability

Minimum suitability = 0

Maximum suitability = 1

Number of suit. categories = 2

Hierarchical Patch Delineation

Gap then Spur = True

Spur then Gap = False

Threshold Perception Values

Landcover gaps removed = 1

Landcover gap--low = 100

Landcover gap--high = 100

# of hierarchical levels = 1

Iterate hierarchy = 0

Landcover spurs removed = 1

Landcover spur--low = 100

Landcover spur--high = 100

# of hierarchical levels = 1

Iterate hierarchy = 0

Minimum area of patch = 0

PatchMorph inputs for the Feather River.

Run name = Fthr2

Input landcover map = Feather_RWFRWS

Landcover map field = YBCU_HAB

Cell size = 10

Density Filter

Density filter = 0

Density neighborhood = 5

Density threshold = 0.25

Hard Barrier

Hard barrier = 1

Hard barrier map = Feather_RWFRWS

Hard barrier map field = WATER

Habitat Suitability

Minimum suitability = 0

Maximum suitability = 1

Number of suit. categories = 2

Hierarchical Patch Delineation

Gap then Spur = True

Spur then Gap = False

Threshold Perception Values

Landcover gaps removed = 1

Landcover gap--low = 100

Landcover gap--high = 100

# of hierarchical levels = 1

Iterate hierarchy = 0

Landcover spurs removed = 1

Landcover spur--low = 100

Landcover spur--high = 100

# of hierarchical levels = 1

Iterate hierarchy = 0

Minimum area of patch = 0
