# Supplementary material for: Current Status of Western Yellow-Billed Cuckoo along the Sacramento and Feather Rivers, California
Source: PLoS One. 2015 Apr 27;10(4):e0125198. doi: 10.1371/journal.pone.0125198 (PMC4411113; doi:10.1371/journal.pone.0125198)
Supplement: S2 Appendix — Historic to current Yellow-billed Cuckoo survey studies along Sacramento and Feather rivers, 1972 to 2013. Information included is the number of cuckoos reported, number of rounds of surveys per breeding season, citation, number of surveyor days (the total number of days a survey was conducted by each person conducting surveys), range of survey dates, our conservative estimate of the number of cuckoo detections (derived by assuming any reported pair was only single a detection of one individual and each unmated detection was one individual), and the number of cuckoos per surveyor day. The 2007 and 2008 study on the Sacramento River only surveyed restored habitat within the Sacramento River National Wildlife Refuge and hence was not used in the analysis of change. (DOCX) [file pone.0125198.s002.docx]

| **Year** | **Reported Results** | **# Survey Rounds** | **Citation** | **Surveyor Days** | **Date Range** | **# Cuckoo Detections** | **Cuckoos/Surveyor Day** |
| --- | --- | --- | --- | --- | --- | --- | --- |
| **Sacramento River** | | | | | | | |
| 1972 | 32 individuals | 1 | [27]Gaines 1973 | 11 | 7/6-8/10 | 32 | 2.909 |
| 1973 | 33 individuals | 1 | [27]Gaines 1973 | 11 | 6/23-8/23 | 33 | 3.000 |
| 1977 | 44 individuals | 1 | [9]Gaines and Laymon 1984 | 20 | 6/5-7/31 | 44 | 2.200 |
| 1987 | 18 pairs, 23 unmated | 3 | [4]Halterman 1991 | 60 | 6/15-8/15 | 41 | 0.683 |
| 1988 | 35 pairs, 31 unmated | 3 | [4]Halterman 1991 | 60 | 6/15-8/15 | 66 | 1.100 |
| 1989 | 26 pairs, 18 unmated | 3 | [4]Halterman 1991 | 60 | 6/15-8/15 | 44 | 0.733 |
| 1990 | 23 pairs, 24 unmated | 3 | [4]Halterman 1991 | 60 | 6/15-8/15 | 47 | 0.783 |
| 1999 | 41 mated, 26 unmated | 3 | [5]Halterman et al. 2001 | 32 | 6/17-8/11 | 67 | 2.094 |
| 2000 | 48 mated, 57 unmated | 3 | [5]Halterman et al. 2001 | 32 | 6/17-8/9 | 105 | 3.281 |
| 2007 | 3 pairs, 12 individual | 3-5 | [10]Hammond 2011 |  | ~6/15-8/31 | 15 |  |
| 2008 | 1 pair, 9 individuals | 3-5 | [10]Hammond 2011 |  | ~6/15-8/31 | 10 |  |
| 2010 | 23 individuals | 4 | [29]Dettling and Howell 2011 | 288 | 6/14-8/17 | 23 | 0.080 |
| 2012 | 8 individuals | 4 | This study | 182 | 6/15-8/16 | 8 | 0.044 |
| 2013 | 10 individuals | 4 | This study | 204 | 6/15-8/16 | 10 | 0.049 |
| **Feather River** | | | | | | | |
| 1972 | 0 individuals | 1 | [27]Gaines 1973 | 3 | 7/12-8/3 | 0 | 0.000 |
| 1973 | 0 individuals | 1 | [27]Gaines 1973 | 2 | 7/11-7/12 | 0 | 0.000 |
| 1977 | 1 individual | 1 | [9]Gaines and Laymon 1984 | 1 | 6/26 | 1 | 1.000 |
| 1987 | 1 pair, 5 unmated | 3 | [2]Laymon and Halterman 1989 | ? | ? | 6 |  |
| 1999 | 0 individuals | 3 | [5]Halterman et al. 2001 | 6 | 6/28-7/26 | 0 | 0.000 |
| 2012 | 0 individuals | 4 | This study | 40 | 6/21-8/3 | 0 | 0.000 |
| 2013 | 0 individuals | 4 | This study | 40 | 6/20-8/9 | 0 | 0.000 |

**S2 Appendix. Historic Yellow-billed Cuckoo Survey Results and Effort for the Sacramento and Feather Rivers.**
